# Cardiac transplant rejection assessment with 18F-FDG PET-CT: initial single-centre experience for diagnosis and management

**DOI:** 10.1186/s41824-024-00191-y

**Published:** 2024-04-19

**Authors:** Owais Dar, Mansimran Singh Dulay, Fernando Riesgo-Gil, Andrew Morley-Smith, Paul Brookes, Haifa Lyster, Alexandra Rice, Stephen R. Underwood, John Dunning, Kshama Wechalekar

**Affiliations:** 1 Royal Brompton and Harefield Hospitals, Part of Guy’s and St Thomas’ NHS Foundation Trust, London, UK; 2 Kings College London, London, UK; 3 Imperial College London, London, UK; 4 Department of Advanced Heart Failure, Transplant and Mechanical Support, Harefield Hospital, Hill End Road, Harefield, UB9 6JH UK

**Keywords:** Cardiac transplant, FDG-PET CT, Rejection, Myocardial avidity, Immunosuppression

## Abstract

**Background:**

Rejection is a major cause of mortality and morbidity in heart transplant (HTx) recipients. Current methods for diagnosing rejection have limitations. Imaging methods to map the entire left ventricle and reliably identify potential sites of rejection is lacking. Animal studies suggest FDG PET-CT (FDG PET) could have potential application in human HTx recipients.

**Methods:**

Between December 2020 and February 2022, all HTx recipients at Harefield Hospital, London, with definite or suspected rejection underwent FDG PET in addition to routine work-up.

**Results:**

Thirty HTx recipients (12 with definite and 18 with suspected rejection) underwent FDG PET scans. Overall, 12 of the 30 patients had FDG PET with increased myocardial avidity, of whom 2 died (17%). Eighteen patients of the 30 patients had FDG PET with no myocardial avidity and all are alive (100%, *p* = 0.15). All patients with definite rejection, scanned within 2 weeks of starting anti-rejection treatment, showed increased myocardial avidity. In 5 cases, FDG PET showed myocardial avidity beyond 6 weeks despite pulsed steroid treatment, suggesting unresolved myocardial rejection.

**Conclusion:**

Preliminary findings suggest FDG PET may have a role in diagnosing cardiac transplant rejection. Future blinded studies are needed to help further validate this.

**Supplementary Information:**

The online version contains supplementary material available at 10.1186/s41824-024-00191-y.

## Background

Approximately 12% of HTx recipients are treated for rejection during the first year of follow-up (Khush et al. 2019). Rejection is the cause of death in 8–10% of cases in the first 3 years (Khush et al. 2019). HTx recipients are usually treated with a combination of tacrolimus or cyclosporine with mycophenolate mofetil immunosuppression. With this combination, the median one-year conditional survival for HTx is 15 years (Khush et al. 2018). During an episode of rejection, immune cells recognise the donor allograft as non-self and become activated. An immunological cascade follows, leading to the recruitment and invasion of inflammatory cells within the heart, resulting in allograft inflammation/myocarditis. Continued rejection leads to cell death, fibrosis, and loss of graft function.

HTx rejection can manifest as a drop in left ventricular ejection fraction, new heart failure symptoms, dysrhythmia, new donor-specific antibodies, or presence of rejection on myocardial biopsy. International clinical standards for diagnosis of HTx rejection rely mainly on echocardiography and endomyocardial biopsy (Costanzo et al. 2010). These clinical standards are recommendations based on expert consensus rather than randomised controlled trials. Between transplant units, there is variation in the diagnosis and treatment of cardiac rejection even though each unit will adhere to clinical standards. Most centres rely on similar diagnostic criteria. The probability of rejection is higher with increased severity or presence of more than one of these factors. The probability of rejection is low in the absence of symptoms, presence of normal left ventricular ejection fraction, normal sinus rhythm, absence of donor-specific antibodies and a normal myocardial biopsy. A non-invasive imaging method to identify areas of rejection has not been identified so far.

Current tests to diagnose rejection have limitations (Kobashigawa 2017). Biopsy is an invasive procedure with a serious risk of complications such as tamponade, death, but also suffers from drawback of sampling error. The interpretation is semi-objective and semi-quantitative (Stewart et al. 2005; Angelini et al. 2011). In contrast, echocardiography provides a global, non-invasive assessment of cardiac function. However, it is also subject to interobserver variability and, on its own and cannot identify areas of rejection. During early onset low-grade rejection, heart function on echocardiography can be normal. The ideal test would be easily accessible, inexpensive, non-invasive, and provide a global assessment of cardiac function. It would also be sensitive for identifying early onset subclinical rejection with high negative and positive predictive values.

FDG PET is a highly sensitive test to identify infection, inflammation and tumours and it is well established in diagnostic and treatment algorithms. Although nonspecific, the inflammatory activity can be seen earliest on PET-CT than any other technique due to the metabolic nature of imaging that changes earlier than structural changes. Because nuclear medicine exams can pinpoint molecular activity, they have the potential to identify disease in its earliest stages. It has a well an established role in assessing response to treatment (Gambhir et al. 2001; Phelps 2000).

FDG PET is well known to be effective in diagnosis and management of inflammatory myocardial conditions such as sarcoidosis, giant cell and eosinophilic myocarditis (Slart et al. 2018; Piriou et al. 2015; Panithaya et al. 2017; Dumarey et al. 2007; Alchammas et al. 2016). Controlled trials using FDGPET to diagnose rejection in animal models of HTx are convincing (Daly et al. 2015; Hoff et al. 1992). To date, FDGPET has not been used for the diagnosis and management of rejection in human HTx recipients. We describe our initial and the first published experience using FDG PET for the diagnosis and management of rejection in human HTx recipients at a single centre, Harefield Hospital, London, UK.

The institutional review board at 'Royal Brompton and Harefield Hospitals Clinical Practice Committee' approved the off-label use of FDG PET imaging in the diagnosis and management of suspected rejection in HTx recipients at Harefield Hospital. The project was registered with the clinical audit committee (project identification number 005091) in accordance with the trust governance policy.

## Methods

### Clinical protocol

Harefield Hospital has over 500 living HTx recipients under follow-up. Weekly multidisciplinary team (MDT) consisting of cardiologists, histopathologists, immunologists, imagers and pharmacists meet to review cases of suspected rejection.

**Fig. 1:**
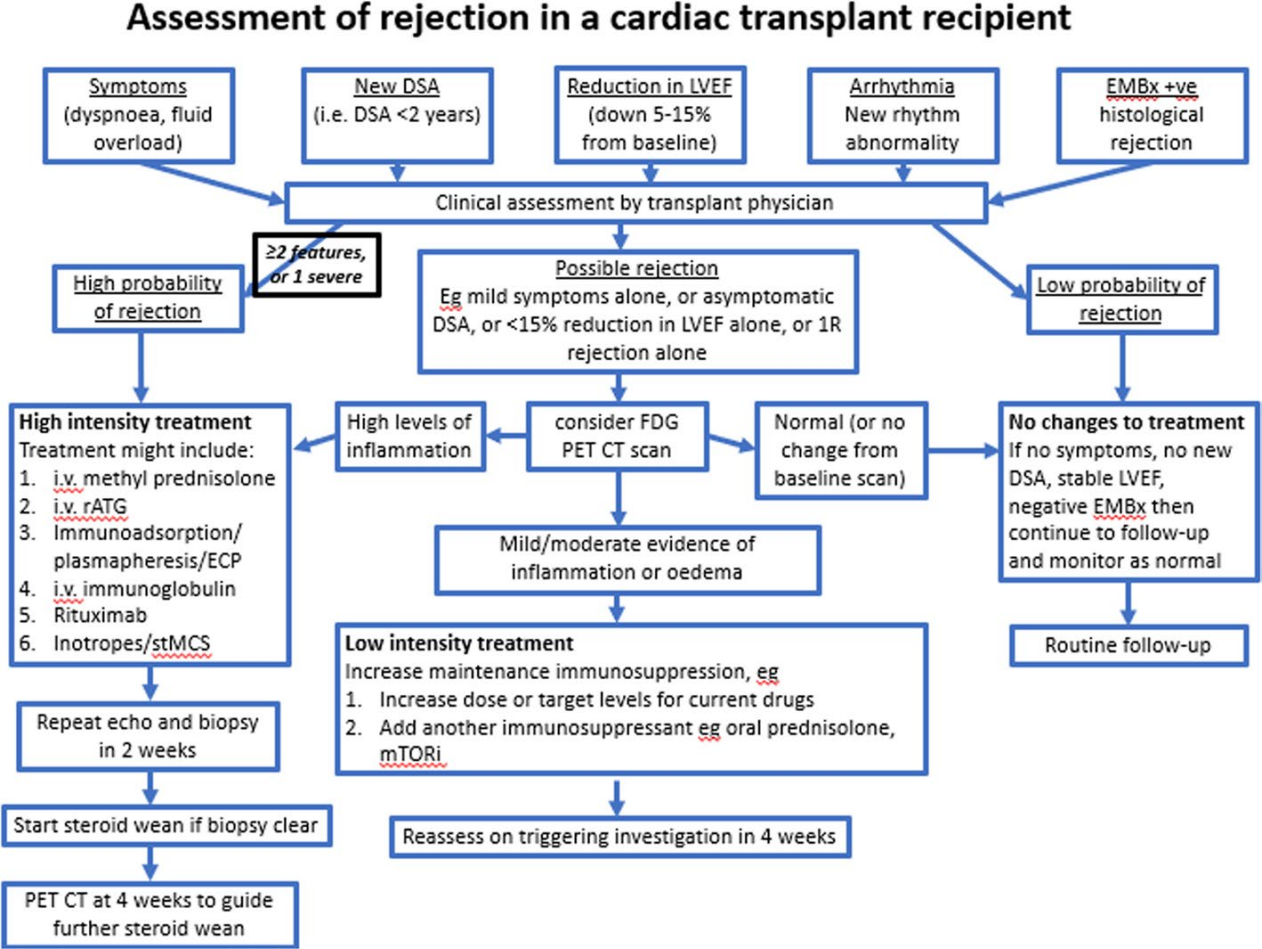
Harefield hospital heart transplant unit consensus rejection assessment and management algorithm

An algorithm to diagnose and manage suspected rejection (Fig. 1) was developed by the MDT. Suspected rejection was defined as a recipient having at least one of the following: (1) new donor-specific antibodies, (2) new symptoms attributable to cardiac rejection, e.g. dyspnoea, (3) new dysrhythmia, e.g. heart block or atrial fibrillation, (4) greater than 5 percentage units fall in left ventricular ejection fraction from baseline, (5) myocardial biopsy suggestive of rejection as defined by international society of heart and lung transplantation.

All patients with clinically suspected rejection at Harefield Hospital HTx unit were included in the study. Patients unable to consent or tolerate FDG PET imaging were excluded from the study.

Suspected rejection episodes were categorised as high probability, possible or low probability on clinical criteria (see Fig. 1).

### FDG PET-protocol

FDG PET avidity within the myocardium can be caused by noncompliance to a carbohydrate free diet pre-scanning, myocardial ischaemia, or myocarditis. To maximise the chance of identifying myocardial damage caused by rejection, all patients had an ischaemia test as part of the PET-CT work-up. All patients were also informed of the importance of dietary compliance to zero-carbohydrate pre-scan.

Patients were advised a zero-carbohydrate diet before the scan followed by 18 h fasting (water only) in order to suppress normal myocardial glucose metabolism (Slart et al. 2021). Imaging was performed using PET-CT scanner with 64-slice CT (Siemens Medical Solutions). A dynamic rest myocardial perfusion scan was first performed using 1110 MBq rubidium-82 to assess myocardial viability and function. Fifteen minutes later, 3 MBq/kg FDG was given I.V. Imaging from eyes to thighs was performed after 90 min. Myocardial scar was classified as mild, moderate, or severe by reduction of rubidium-82 counts of 49–40%, 39–30% and < 30% of maximum myocardial counts, respectively. Myocardial FDG activity was classified as absent, low (SUVmax 2.5–3), medium (SUVmax 3–5) or high (SUVmax > 5).

### Treatment protocol

Recipients with a high probability of rejection were treated with iv methylprednisolone 1 g daily for 3 days. Patients with antibody-mediated rejection were also treated with immunoadsorption, immunoglobulins, and rituximab. Following the initial treatment phase, recipients were discharged home with a tapering dose of oral steroids to be maintained on prednisolone 15 mg daily. Two weeks later, the prednisolone dose was further tapered to 10 mg daily if symptoms and echocardiogram were stable. Four weeks after discharge, FDG PET and myocardial biopsy were performed to determine whether the steroid dose could be completely weaned.

Recipients with a low probability of rejection were not treated as above and were followed up in the clinic for surveillance.

Recipients with only mild degrees of one of the above features were deemed to have possible rejection and underwent FDG-PET for further categorisation. Recipients with FDG-avid myocardium were deemed to have a high probability of rejection and were treated as above. Recipients without FDG-avid myocardium were treated less aggressively with oral immunosuppression optimised to target ranges and enhanced monitoring.

### Aims and objectives

As per the recommendations of the institutional clinical practice governance committee, a review of the FDG PET CT HTx rejection service was conducted. The aims and objectives of the review were as follows: (i) to identify whether FDG PET can detect cardiac rejection in patients deemed to have high probability of rejection from clinical criteria, (ii) to identify whether FDG PET can appropriately classify patients with possible rejection, (iii) to identify whether FDG PET can help with safe steroid weaning following high-dose intravenous methylprednisolone treatment for rejection and (iv) to identify if FDG avidity is associated with patient outcomes.

### Statistical analysis

Descriptive data were represented as frequencies, percentages, and medians, and Fischer’s exact test was used to assess differences in mortality between FDG avid and non-avid groups.

## Results

### Patient characteristics

Thirty HTx patients underwent FDG PET for the assessment of rejection between December 2020 and February 2022. Twenty-two were male median age was 49 years (range 19–79). Median time after transplantation was 10 years (range 1–31). Median follow-up time was 9 months (range 1–16). Ten patients had donor-specific antibodies, of which 8 were long-standing. Twelve patients had an eGFR between 30 and 59 ml/min/1.73 m^2^. Four patients had severe graft coronary disease and 20 were on antihypertensive medication. Ten patients were obese with BMI > 30 kg/m^2^. Twenty-three patients received CNI therapy in combination with MMF for immunosuppression. Twenty-five patients received statin therapy. Baseline characteristics are summarised below in Table 1.

**Table 1:** Baseline demographics

Demographic categories	Frequency	Percentage
*Gender*		
Female	8	27
Male	22	73
*Age*		
18–24	5	17
25–34	5	17
35–44	3	10
45–54	5	17
55–64	4	13
65 +	8	26
*Years post-transplant*		
< 1	1	3
1–5	9	30
6–10	7	23
11–15	3	10
16–20	3	10
21–25	6	20
+ 25	1	3
*Donor-specific antibody positive*		
total	10	33
> 3 years	8	27
Diabetes	2	7
*Renal dysfunction*		
Kidney transplant	3	10
eGFR > 89	3	10
eGFR 60–89	12	40
eGFR 45–59	10	33
eGFR 30–44	2	6
eGFR 15–29	0	0
eGFR < 15	0	0
*Graft vascular disease*		
None	8	27
Mild	18	60
Previous coronary stents	4	13
Hypertension	20	67
*Body mass index*		
18.5–24.9	6	20
25–29.9	14	47
30–39.9	10	33
*Immunosuppression agents*		
Tacrolimus and MMF	18	60
Ciclosporin and MMF	5	17
Calcineurin inhibitor and Azathioprine	4	13
Calcineurin inhibitor monotherapy	2	7
Sirolimus and MMF	1	3
Statin	25	83
Ezetimibe	2	7

### Outcomes

**Fig. 2:**
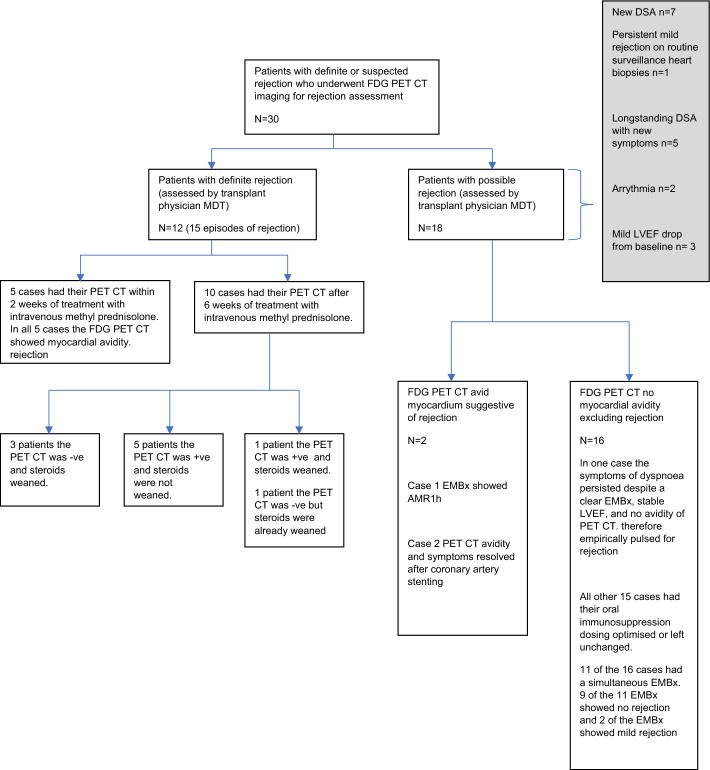
Study flow chart

The outcomes of this study are summarised according to each of our study aims (see Fig. 2 for an overview of the study and outcomes).

1. If FDG PET can detect cardiac rejection in patients deemed to have high probability of rejection from clinical criteria:

Twelve patients were deemed to have definite rejection by previously defined clinical criteria. They were admitted for 3 days of high-dose iv methylprednisolone. Three patients had 2 separate episodes of rejection and the remaining 9 patients had one episode of rejection giving a total of 15 separate episodes of definite rejection. In five rejection episodes, FDG PET was performed within 2 weeks of steroid pulsing and all patients demonstrated significant myocardial FDG avidity.

Three out of these 5 patients had mild rejection on the heart biopsy, one had no evidence of rejection and one was unable to tolerate the biopsy. Three out of the five patients had high levels of avidity, one had medium level of avidity and the remaining one had low level of avidity on the PET CT.

Four of the five patients had no scar on the PET CT. One patient had an LV apical scar amounting to 4% of the myocardium.

2. To identify whether FDG PET can appropriately classify patients with possible rejection:

Eighteen patients were deemed to have possible rejection by clinical criteria. Seven of these had asymptomatic new donor-specific antibodies, 1 had asymptomatic mild rejection on biopsy despite normal LV function, 5 had new symptoms in the context of long-standing (more than 3 years) donor-specific antibodies and normal LV function, 2 had new dysrhythmia with normal function and biopsy, and 3 had an asymptomatic fall in LVEF.

**Fig. 3:**
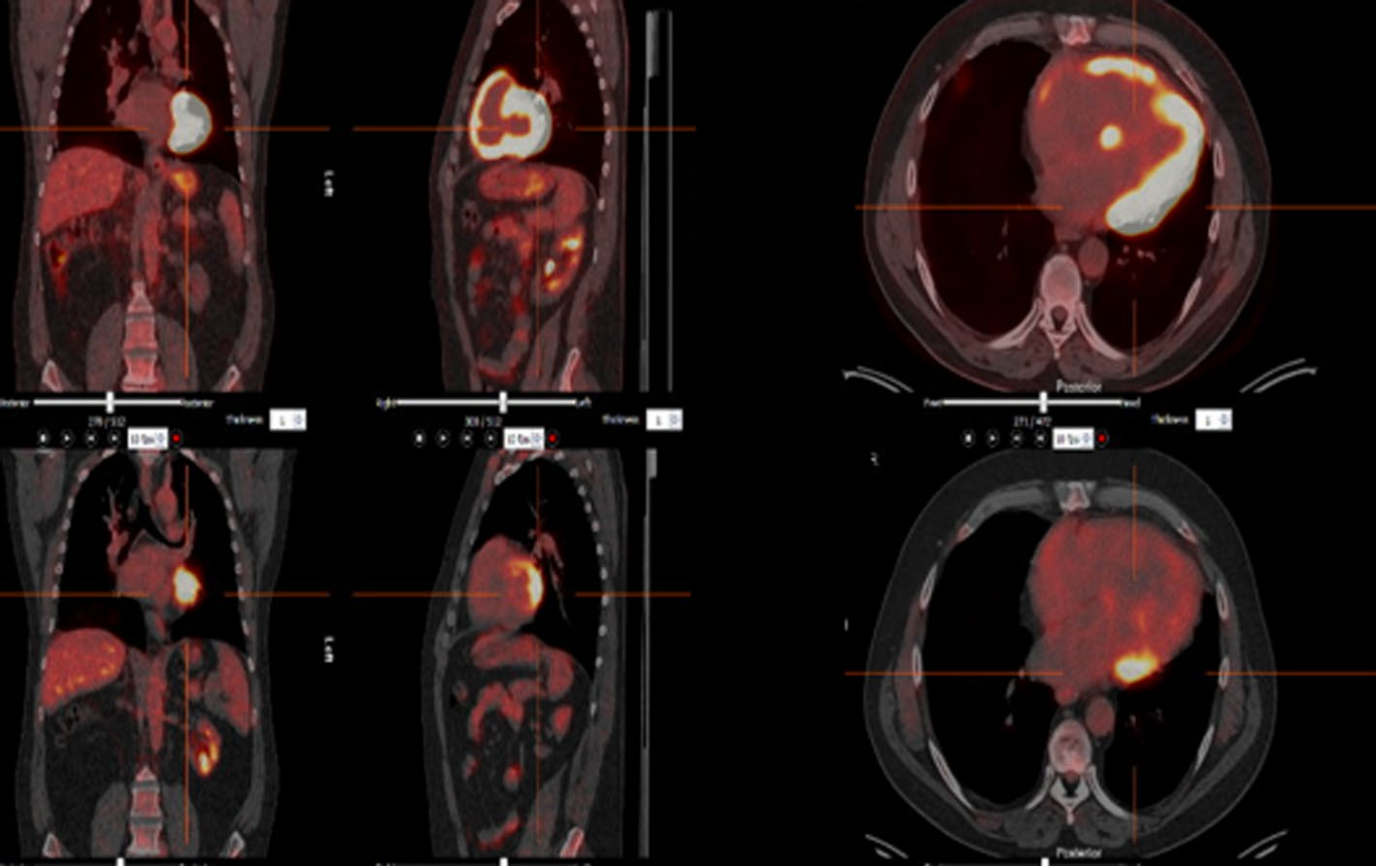
The upper row shows intensely active myocardial inflammation in much of the LV sparing parts of the septum (usual site for EMBx), free wall of right ventricle and low-grade inflammation in the right atrium. The lower row shows images after treatment. There is remnant inflammation in the basal anterolateral wall but almost complete resolution elsewhere

Of these 18, only 2 had myocardial FDG avidity. One of the two patients was asymptomatic with normal LVEF but had new donor-specific antibodies and Grade 1 antibody-mediated rejection on biopsy. He was treated with iv methylprednisolone, and repeat PET-CT fifty-two days after treatment showed near complete resolution of myocardial FDG (Fig. 3). The second patient had chest pain and dyspnoea despite unchanged LVEF of 31%. FDG PET was consistent with either rejection or ischaemia and invasive angiography showed left anterior descending and circumflex coronary stenoses, leading to percutaneous coronary stenting. Repeat PET-CT showed resolution of myocardial inflammation and the patient’s dyspnoea and chest pain resolved.

In the remaining 16 patients with possible rejection, there was no myocardial FDG. In 15 of these, oral immunosuppression was optimised or unchanged and 1 of the 16 had persistent dyspnoea despite normal LV function, and biopsy. He was pulsed empirically with iv methylprednisolone and oral diuretics with some improvement. Eleven of the 16 underwent biopsy of which 9 showed no rejection in keeping with PET-CT findings.

3. To identify whether FDG PET can help with safe steroid weaning following high-dose intravenous methylprednisolone treatment for rejection.

In 10 patients, FDG PET was performed 6 weeks after steroid pulsing. Three of these had no myocardial FDG avidity and oral steroids were weaned. Five had myocardial FDG avidity and the steroids were not weaned. One of these 10 had myocardial FDG avidity but myocardial biopsy and LV function were normal and so steroids were weaned, but the patient died suddenly at home. In the last of these 10, there was no myocardial FDG but steroids had already been weaned and rejection was considered unlikely with no symptoms and normal LV function.

4. To identify if FDG avidity is associated with patient outcomes.

Of all 30 patients, 12 had abnormal myocardial FDG, of whom 2 died and 18 patients had no myocardial FDG uptake and are alive at follow-up, *P* = 0.15 (see Table 2, and Additional file 1: supplementary data for a more detailed case history of the 2 patients who died).

**Table 2:** Number of patients alive and dead categorised according to myocardial FDG PET CT avidity

	FDG PET CT shows myocardial avidity	FDG PET CT shows no myocardial avidity
Alive	10	18
Dead	2	0

## Discussion

FDG PET is routinely used for the diagnosis and monitoring of patients with autoimmune myocarditis, such as eosinophilic and giant cell myocarditis (Slart et al. 2018; Piriou et al. 2015; Panithaya et al. 2017; Dumarey et al. 2007; Alchammas et al. 2016), and for myocardial inflammation in patients with sarcoidosis. Animal studies of HTx suggest that it is a reliable and quantitative technique for monitoring allograft rejection with the potential for application in humans (Daly et al. 2015; Hoff et al. 1992). To our knowledge, this is the first published report on the use of FDG PET for the diagnosis and management of rejection in humans.

Our data suggest that FDG PET can reliably identify definite rejection, as determined by established techniques. All of our patients with definite rejection who underwent FDG PET within 2 weeks of anti-rejection treatment had FDG-avid myocardium using a dietary regime to suppress normal myocardial glucose metabolism.

An ideal rejection test should be able to monitor degree of rejection and show response to therapy. Such a test would allow the safe tapering of immunosuppression intensity at an appropriate level to control rejection but limit the adverse effects of immunosuppression such as infection, malignancy, and renal impairment. Before the introduction of FDG PET, we routinely weaned steroids following rejection treatment 2 weeks after pulsed steroids if LV function was stable. However, our experience with FDG PET suggests that weaning should be delayed beyond 2 weeks to a maximum of 6 weeks as guided by PET-CT. FDG PET could be very useful for ongoing monitoring of response to medical therapy for rejection but needs further evaluation in controlled blinded studies.

We have also observed a clinical but not statistically significant association between myocardial FDG avidity and survival. Both patients who died despite aggressive treatment for rejection had persistent avidity on their PET-CT scan. Whilst this could represent ongoing rejection, it is also possible that it could represent ongoing microvascular ischaemia undetected by coronary angiography. Treatment options for microvascular ischaemia are very limited. Regardless, PET-CT may be able to identify a cohort at high risk of death.

In our cohort, FDG PET was well tolerated by all patients. None of the patients failed to complete the scan. No immediate adverse effects were noted. The overall radiation dose per patient was 11.3 millisievert which was equivalent to 4.5 years of average natural background radiation of UK. An unexpected benefit of performing the PET-CT scan was the identification of additional pathology in two cases. An adenocarcinoma of the lung and a cryptococcal abscess were identified in two separate cases which led to early successful diagnosis and treatment.

The implication of these incidental findings begs the question of using non-invasive imaging to screen for malignancies or infection by heart transplant community. The risk of malignant tumours after HTx is significantly higher than the general population. Due to a lack of sensitivity, specificity and high cost, screening for subclinical malignancy using tumour markers has been shown to be of limited benefit post heart transplantation. The use of PET-CT as a screening tool in HTx recipients is yet unknown (Nägele et al. 1999).

Uptake of FDG in heart can be multifactorial. Most importantly, patient preparation to suppress myocardial glucose utilisation is essential. Although there is no conclusive test to prove achievement of such a state, detailed instructions, diet history and diffuse intense pattern of uptake in the myocardium can help to characterise the failure of protocol. Ischaemic myocardium can also demonstrate FDG uptake. Cardiac transplant patients are known to have advanced coronary vasculopathy which can manifest as epicardial coronary and or microvascular disease. It is considered as a continuum of rejection. Although we screened patients for epicardial coronary disease, we have not been able to exclude microvascular disease conclusively in this group of patients.

In our institution, the practicalities of getting a PET-CT require patients to travel to another location outside the transplant unit. PET-CT scanners are not widely available and are mainly used for oncological imaging. This maybe a reason for its limited utilisation. Commissioners and health service organisations would need supportive cost effectiveness data for PET-CT imaging to become a clinical standard for the diagnosis of rejection.

Other comparable non-invasive imaging techniques such as CMR have also been studied in diagnosing rejection in HTx recipients. These studies suggest CMR has an 80–90% sensitivity in picking up moderate cardiac rejection as defined by cardiac biopsy. Although CMR may have a role in rejection diagnosis, the results of these studies should be interpreted cautiously as they are limited in their design. The studies were retrospective, not blinded, used differing CMR parameters to identify rejection, had small numbers, and were conducted in single centres (Han et al. 2021).

One single-centre study randomised 40 patients to CMR surveillance for rejection versus EMBx surveillance in the first-year post HTx. The results of this study are promising, suggesting CMR guided surveillance is not inferior to the current standard of care of EMBx surveillance. However, this single centre, small sized study used a definition of rejection that was reliant on biopsy alone. The reproducibility, long-term impact on graft survival and function still needs to be proved (Anthony et al. 2022).

## Limitations

This study is not a blinded trial but rather a report on a clinical off-label experience of the use of PET-CT. The clinical protocol was developed by our heart transplant team and approved by the Harefield Hospital clinical practice committee who oversee the governance and approval of the off-label use of techniques and medical interventions. The findings of the study should be interpreted in this context and are more hypothesis generating than clinical proof.

There is no gold standard test or definition for cardiac rejection. We used a clinical standard definition of cardiac rejection which incorporated transplant physician MDT opinion, EMBx, echocardiography, clinical symptoms, ECG, and presence of donor-specific antibodies to determine the presence or absence of rejection. We developed a consensus document on the diagnosis and management of rejection (see Fig. 1) and decided on all suspected cases of rejection in an MDT setting as described above. Other studies have used EMBx as their gold standard definition of rejection. We intentionally avoided this to reflect real world practice and the clinical practice of our unit. An advantage of this meant that we could recognise rejection that was biopsy negative and diagnose rejection when biopsies were inconclusive or inadequate.

This study found the presence of persistent inflammation on FDG PET in some patients 6 weeks following treatment for rejection. We used the presence of persistent inflammation on FDG PET to help guide the decision to wean steroids to avoid the long-term adverse effects of steroid use. In contemporary practice, the decision to wean or continue steroids is based on standard diagnostics, i.e. echocardiography, EMBx and clinical symptoms. Using PET-CT to determine weaning of steroids reflects clinical practice in management of active cardiac sarcoidosis and other autoimmune myocarditic conditions. However, further studies are needed to clarify other reasons for persistent FDG uptake such as fasting failure or ischaemia which may need more prolonged dietary preparation or revascularisation and not necessarily need further immunosuppressive treatment. Our findings should be interpreted in this context and do not represent conclusive proof but rather should be viewed as hypothesis generating.

## Conclusion

This is the world’s first report on the clinical use of PET CT imaging for the diagnosis and management of rejection in human heart transplant recipients. In our preliminary observational study, FDG PET appeared to be a promising new tool for non-invasive global assessment and management of cardiac rejection. Whilst the results of our novel study are encouraging, they should be reviewed with some caution due to the small study size, single-centre experience, and unblinded study design. In the future, larger, multicentre, and blinded diagnostic accuracy study needs would better evaluate the role of this technique.

### Supplementary Information

**Additional file 1**: Supplementary data providing a clinical synopsis of the 2 patients who died.

## Data Availability

Datasets from which conclusions within this manuscript were drawn are presented in this article and its associated figures/tables/supplementary data (all available within this manuscript). Any additional data that may be required will be made available on request.

## Abbreviations

BMI: Body mass index
CMR: Cardiac magnetic resonance
CNI: Calcineurin inhibitor
DSA: Donor-specific antibodies
ECP: Extra corporeal photopheresis
EMBx: Endomyocardial biopsy
FDG PET: ^18^F Fluorodeoxyglucose positron emission tomography and computed tomography
HTx: Heart transplant
I.V: Intravenous
LV: Left ventricle
LVEF: Left ventricular ejection fraction
MDT: Multi-disciplinary team
Kg: Kilogram
Mg: Milligram
MBq: Megabecquerel
MMF: Mycophenolate mofetil
mTORi: Mechanistic target of rapamycin inhibitor
−ve: Negative
+ve: Positive
RATG: Rabbit anti-thymocyte globulin
stMCS: Short term mechanical circulatory support
SUVmax: Standardised uptake value maximum
UK: United Kingdom
