# Supplementary material for: Cardiac transplant rejection assessment with 18F-FDG PET-CT: initial single-centre experience for diagnosis and management
Source: EJNMMI Rep. 2024 Apr 19;8(1):9. doi: 10.1186/s41824-024-00191-y (PMC11026309; doi:10.1186/s41824-024-00191-y)
Supplement: Supplementary file 1 — Additional file 1: Supplementary data providing a clinical synopsis of the 2 patients who died. [file 41824_2024_191_MOESM1_ESM.docx]

**Supplementary data**

Of the 2 patients who died, the first was a 33-year-old gentleman who was 3 years post re-do HTx. Two years after his re-do HTx, due to symptoms of new onset breathlessness and a fall in LVEF from 67% to 51% on echocardiogram, he was treated for rejection with iv methyl prednisolone. Cardiac biopsy showed no evidence of rejection and invasive coronary angiogram showed non flow limiting mild coronary stenoses. Repeat FDG PET CT scan 3 months after treatment for rejection showed improving (SUV 24 to SUV of 7.5) but ongoing high levels of myocardial avidity. Treatment with steroids therefore was continued and he was managed with tacrolimus, MMF 1 gram twice daily, and prednisolone 10mg daily. A month later he was re-admitted with worsening dyspnoea and a significant drop in echocardiographic LVEF to 31%. He had a repeat invasive angiogram which showed worsening severe mid-vessel left anterior descending artery stenosis and a proximal severe stenosis in an obtuse marginal artery. He had an angioplasty and stent to the left anterior descending and obtuse marginal arteries. Heart biopsy confirmed grade 1R cellular rejection. He was treated with iv methyl prednisolone 1g for 3 days. Five days after the revascularisation he died unexpectedly whilst on the ward from a cardiac arrest despite attempts to resuscitate him. The documented cause of death was cardiac transplant related coronary artery disease secondary to acute on chronic rejection following redo HTx.

The second patient was a 20 year old lady who had a HTx for restrictive cardiomyopathy at the age of 5. She developed new onset donor specific antibodies at the age of 20 and was admitted to hospital with breathlessness. An echocardiogram showed a significant drop in LVEF from 72% at baseline to 45% with a significant pericardial effusion. EMBx was inconclusive. She was treated for rejection with iv methyl prednisolone, immunoglobulin, rituximab and immunoadsorption therapy. Her pericardial effusion was drained. A CT coronary angiogram showed no evidence of coronary disease. She was discharged home with an LVEF of 55%. Two months later EMBx was performed which was non diagnostic and inconclusive. At the same time a PET CT showed high myocardial avidity (SUV 9.5) within the left ventricle and right ventricles so her steroids were not weaned. A EMBx 2 months after this showed no rejection. A month later during clinic review she was noted to have recurrent worsening breathlessness. She underwent another cycle of rejection treatment with immunoglobulins, rituximab and immunoadsorption therapy. She was discharged home with an LVEF of 53% and improved symptoms with a plan to have further cycles of immunoadsorption therapy. Her immunosuppression at the time was tacrolimus, myfortic 360mg twice daily, and prednisolone 10mg daily. Unfortunately, she died suddenly at home. Cause of death was rejection post HTx.
